# Evaluating the impact of a longitudinal mentorship intervention on the documentation of maternal vital signs in Blantyre district, Malawi

**DOI:** 10.1186/s12884-025-08493-0

**Published:** 2025-12-30

**Authors:** Ashley Mitchell, Nelson Ntemang’ombe Mwale, Luseshelo Simwinga, Oveka Jana, Miranda Rouse, Kimberly Baltzell, Alden Hooper Blair

**Affiliations:** 1 UCSF Global Action in Nursing (GAIN), San Francisco, CA USA; 2 Institute for Global Health Sciences, University of California (UCSF), San Francisco, CA USA; 3 GAIA Global Health, Blantyre, Malawi; 4 Department of Family Health Care Nursing, University of California, San Francisco, CA USA; 5 Malawi Ministry of Health, Lilongwe, Malawi

## Abstract

**Background:**

Staff shortages, insufficient training and support, and high patient caseloads limit maternal quality of care (QoC) and influence poor documentation of vital signs and labor progress in Malawi. Aware that this limits providers’ ability to anticipate or manage complications, we explored the impact of a longitudinal multipronged intervention on the documentation of maternal vital signs at key clinical times during childbirth to identify targeted opportunities for improvement.

**Methods:**

We conducted a retrospective quantitative analysis of maternal charts from two primary health centres in Blantyre district in Malawi to assess for differences in the documentation of vital signs established in the WHO Safe Childbirth Checklist (SCC). The intervention consisted of short course training followed by 12 months of bedside mentorship. Bivariate and multivariate analyses assessed differences in the recording of each vital sign both pre- and post-intervention as well as according to availability of the appropriate device for that vital sign.

**Results:**

A total of 271 maternal charts—96 from the pre-intervention period and 175 from the post-intervention period—were analyzed and found to have recorded between 2% and 52% of key maternal vital signs at the SCC-designated times. Post-intervention charts showed a statistically significant (*p <* 0.05) increase in the documentation of heart rate/pulse and blood pressure both upon admission and immediately postpartum, though not at the time of active childbirth. Additionally, while few maternal charts included all vital signs, there was a significant increase in the number of vital signs recorded between the pre- and post-intervention periods. A sub-analysis explored the impact of the availability of key medical devices on documentation during the post-intervention period and found that the recorded availability of thermometers and blood pressure cuffs were not significantly associated with whether temperature or blood pressure was recorded, respectively. However, at admission, significantly more vital signs were recorded when all a centre’s medical devices were consistently available.

**Conclusion:**

A deeper exploration into which strategies are most effective for vital sign measurement and how it affects QoC indicators is warranted. Meanwhile, continuing and expanding training followed by supportive mentorship will be key to making sustainable maternal QoC improvement.

**Supplementary Information:**

The online version contains supplementary material available at 10.1186/s12884-025-08493-0.

## Background

Quality of care (QoC)—the extent to which health services are safe, effective, and person-centered across the patient care continuum—continues to be a strong indicator of maternal and neonatal health globally [1–3]. Improving QoC is critical to prevent and address adverse conditions as evidence has demonstrated that access to, and utilization of, care throughout pregnancy and the perinatal period are insufficient to improve health outcomes on their own [1, 4]. According to the World Health Organization (WHO) and the Institute of Medicine (IOM), quality maternal and newborn care entails providing healthcare meets QoC standards and is timely, efficient, integrated, and equitable [2, 5]. Many of these ideals are considered to be met when a patient is provided with the contextually-relevant “gold standard” of care, which can only be achieved with the appropriate community-, centre-, and district-level physical and human infastructure [1]. With appropriate infrastructure in place, the prevention and management of perinatal complications including hemorrhage, hypertensive disorders, and asphyxia, among others, could significantly reduce morbidity and mortality for women and neonates [4, 5]. 

Particularly in low-resource settings, such as Malawi where this study took place, efforts to improve QoC tend to be fragmented and underutilized. This is in part because robust data are needed—beyond indicators routinely required by national health information systems—to track, inform, and ultimately improve care [5]. In these settings, staff shortages, insufficient training and support, and high patient caseloads further limit the possibility of delivering or evaluating QoC [6, 7]. While skilled attendance of births has increased notably in Malawi, with up to 91% of deliveries occurring in health centres, several bottlenecks remain which challenge care quality [8, 9]. Human resource and supply shortages coupled with limited infrastructure including physical space, personnel supervision and support, and robust referral systems, results in deficits across the care continuum [6, 7, 10]. These challenges contribute to poor documentation of both vital signs and labor progress, limiting providers’ ability to anticipate or manage complications [11, 12]. 

Malawi’s national leadership has demonstrated that quality of centre-based care is a priority. The government both supports research on the topic and recently implementing an adapted WHO Safe Childbirth Checklist (SCC) tool to assess maternal care with the support of multidisciplinary partners [13, 14]. Still, findings show clear challenges and areas for improvement. A nationally representative assessment of centre delivery care demonstrated that peripheral health centres lag behind larger health care centres and hospitals in QoC [9]. Another study across five districts demonstrated that less than half of centres met the QoC indicators for emergency obstetric care, maternity ward staffing and triaging, supply of essential drugs and equipment, diagnosis and management of eclampsia and pre-eclampsia, and management of postpartum hemorrhage [13]. This suggests a need for additional investigation into the proximal and distal drivers of poor QoC in the country.

## Methods

This study aimed to explore the impact of a longitudinal multipronged intervention on the provision and documentation of maternal vital signs at each SCC Pause Point. We conducted a retrospective quantitative analysis of maternal charts from primary health centres in Blantyre district in Malawi between January 2018 and September 2023. In addition to summarizing progress toward implementation of the WHO SCC, we identified opportunities for further improvement.

### Setting and participants

Since 2017, the University of California San Francisco (UCSF) Global Action in Nursing (GAIN) project has collaborated with local partners across four countries, including Malawi [15]. With a vision to prevent maternal and neonatal complications and mortality, GAIN partners with local government and non-profit organizations to ensure nurses and midwives are well-equipped with adequate knowledge, skills, and attitudes to support women in childbirth. Since 2019, GAIN has worked with seven peripheral health centres in Blantyre district, two of which—the foci of this study—were added in February 2021 [16]. All partnering health centres were selected by local Malawi government officials based on their high caseload of maternal and neonatal patients.

Blantyre district is a microcosm of the national and regional QoC trends described earlier, with “higher quality” centres—according to criteria established by the Malawi Service Provision Assessment (SPA)—concentrated around the tertiary care centre, Queen Elizabeth Central Hospital (QECH), compared to more rural sites [9]. SCC vital sign measurement was determined to be a priority through consultation with health centre leaders and the Blantyre District Health Office (DHO). This was achieved through comprehensive analyses of maternal patient charts by a research midwife (author NM) before and after the implementation of an intervention to improve QoC in maternal and neonatal care. While most QoC frameworks incorporate both the provision and experience of care, this study focuses on the former, aligning with a priority to improve health outcomes [4]. 

### The intervention

The intervention in Blantyre district included a package of intensive short-course trainings (emergency obstetric and newborn care, nurse leadership, quality improvement approaches, etc.), 12 months of longitudinal bedside mentorship by site-specific expert nurse midwives, and data strengthening activities [16]. This included the rollout of the Malawian adapted WHO SCC “Pause Points” with foci into QoC: (1) before birth upon admission; (2) just before pushing or cesarean delivery; (3) within one hour after birth; and (4) prior to discharge [17]. Simultaneously, the intervention supported the provision of critical medical devices to measure maternal vital signs (stethoscopes, thermometers and blood pressure (BP) cuffs) and worked with centre leads and the District Nursing and Midwifery Officer (DNO) to ensure they are repaired as needed. As part of regular ongoing activities, a study team midwife mentor (author: LS) rotated between the centres to assess availability of functioning medical devices used for vital sign measurement and critical to QoC.

### Methodological approach

In 2024, our team conducted a exploration of the two centres in this study as well as other local centres utilizing data from District Health Information System 2 database (DHIS2) [18]. While this study revealed promising results related to the intervention, including increased reporting of complications and reduced missingness in the data, it faced limitations in its evaluation. Specifically, we were limited due to the known underreporting in national-level data where prior research in Malawi has suggested that up to a third of key data, such as maternal complications, may be missing from data registries including DHIS2 [12]. It was also limited in its ability to assess patient-level characteristics of the intervention due to the DHIS2 being drawn from amalgamated monthly reports. The present study builds upon this work by collecting primary data from two key centres at the patient level. This approach also allowed for the collection of data related to relevant centre supplies.

**Fig. 1:**
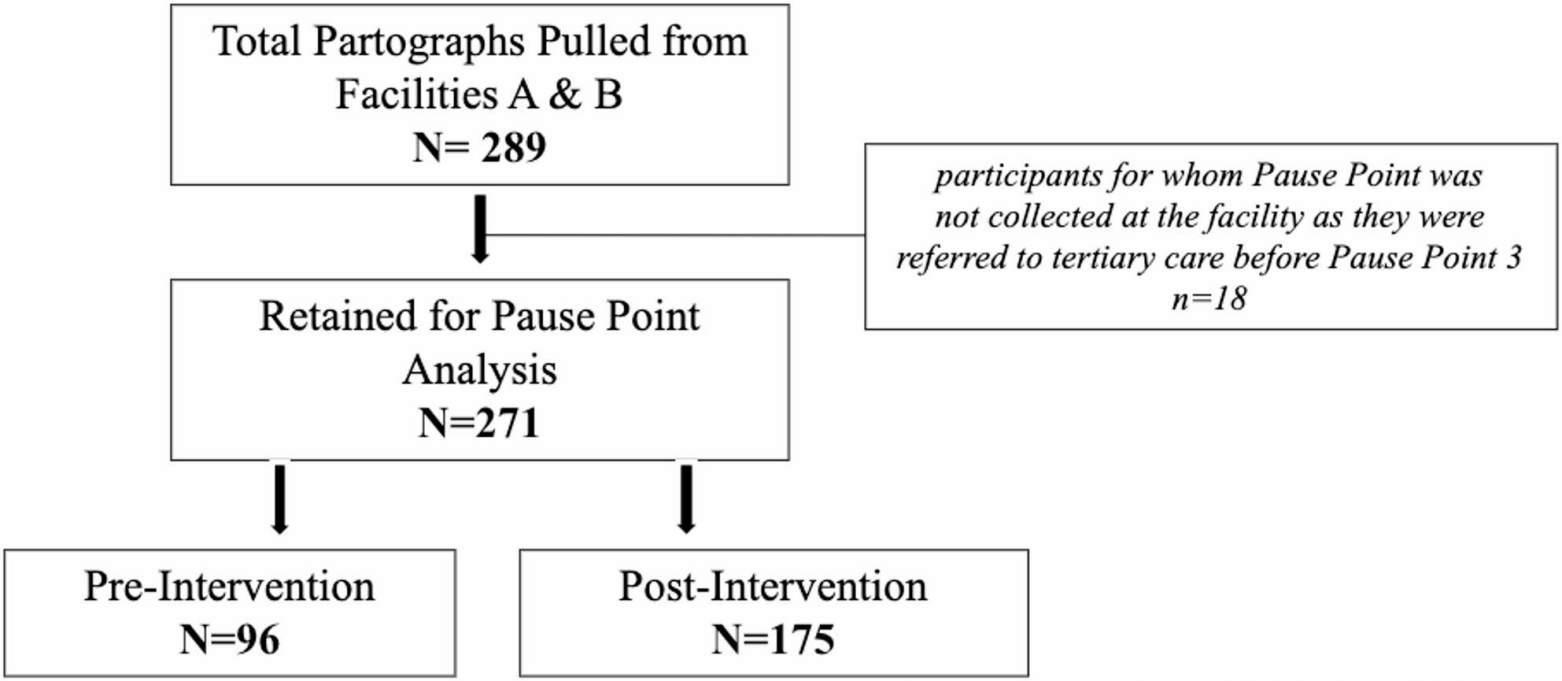
Flowchart depicting Pause Point Analysis and Post-Intervention samples

Maternal charts were pulled from the two primary health centres added during the 2021 expansion (referred to as “Centre A” and “Centre B” throughout, to avoid stigmatizing a given centre and focus on the results) as well as from QECH—the referral site for obstetric complications across Blantyre district (Fig. 1). The latter was included as patients with complications who are referred to QECH for higher level care, as patients who are referred travel with their charts. Of note, Centre A is slightly larger and considered peri urban, located about 18 km from QECH, while Centre B is more rural and situated more than 30 km away. The data collection focused on establishing a baseline of care in a ‘pre-intervention’ period from January 2018 to February 2021 and a ‘post-intervention’ period of March 2021 and after.

Deidentified data focusing on key maternal vital signs (temperature, pulse/heart rate, and BP) were recorded across each of the SCC Pause Points (Appendix I).These were entered into CommCare, a customizable digital software platform for offline data collection, by a Malawian research nurse midwife and GAIN midwife mentor (author: NM) [19]. An average availability score between 0% and 100% was computed to represent the availability of thermometers, stethoscopes, and BP cuffs each month. These data were used to assess the impact of supplies on the provision and reporting of care in the post-GAIN period. To ensure the reliability of the results, an a-priori sample size calculation determined a need for at least 95 charts in each of the pre- and post-intervention periods to achieve a minimum power of 80%, two-sided alpha of 0.05, and clinical effect size of 10% deemed ‘clinically significant’ by the study team midwife mentors (author: LS, NM).

### Data analysis

Maternal chart data were exported as an Excel spreadsheet for initial cleaning and then uploaded to the statistical analysis program R version 4.1.0 (2021-05−18) for analyses [20]. The recording of key maternal vital signs was summarized for each Pause Point and centre. Initial summaries revealed that less than 3.0% of records included the fourth Pause Point (prior to discharge) and so analyses focused a subset of data that included Pause Points one through three accordingly. An *Average Vital Statistics Recorded Score* of zero (no vital signs recorded) to three (all vital signs recorded) was computed for each chart at each Pause Point. Analyses then explored SCC documentation in relation to the intervention (pre/post), the availability of medical devices, and by each health centre.

Summary descriptive statistics were followed by bivariate Kruskal Wallis and Fisher’s exact tests to analyze differences in vital statistics documentation between centres as well as availability of functioning medical devices between centres. Multi-variable logistic regressions assessed differences in the recording of each vital sign both pre- and post-intervention and according to availability of the appropriate device for that vital sign (i.e., impact of availability of functioning thermometer(s) on the recording of temperature). Across tests, a *p-value* (< 0.05 considered statistically significant), point estimates, and 95% confidence intervals (95%CI) were calculated to show both strength and directionality of associations. Finally, a sub-analysis of post-intervention data was conducted to summarize device availability (% of time functioning devices were present with SD to show variability) and analyze vital signs at the centres by the overall level of supplies available.

### Ethical considerations

The study was approved by the QECH research committee, the National Health Sciences Research Committee (NHSRC) (19/03/2210), and UCSF Human Research Protection Program (HRPP) (18–26842). The study proposal, GAIN activities, and results are shared on an ongoing basis with members of the Blantyre DHO as well as the health centres’ and QECH maternal health providers. Funding for this project was generously provided by the Wyss Medical Foundation.

### Researcher word choice

In instances where several words could be used to convey the same concept, we have intentionally chosen terms commonly used in Malawi where the study takes place. The term “health centre” and “centre” refers to the primary health care facilities from which data were collected. Additionally, while the term ‘birthing person’ increasingly describes those with a capacity to give birth, in this paper we defer to gendered language such as ‘woman’ and ‘maternal’ aligning with Malawian reporting. Finally, we use “site-specific expert nurse midwives” to refer to lead providers stationed within one centre and “study team midwife mentors” to refer to cross-site mentors who rotate between centres to provide high-level support while leading research and quality improvement activities.

## Results

A total of 271 maternal charts—163 from the larger peri-urban Centre A and 108 from the rural Centre B—were analyzed, 96 from the pre-intervention period and 175 from the post-intervention intervention period. Overall, charts were found to have recorded between 2% and 52% of key maternal vital signs across all Pause Points (Table 1). There were only two instances in which it was unclear within the patient’s chart whether a vital sign was taken at a particular time and was therefore marked “Unknown.” The first and third Pause Points saw better performance than the second Pause Point, where temperature, heart rate/pulse, and BP were each recorded less than 20% of the time. The only measures recorded consistently in over 50% of charts were heart rate/pulse and BP at the first Pause Point.

Calculation of *Average Vital Statistics Recorded Scores* ranged from of zero (no vital signs recorded) to three (all vital signs recorded) for all Pause Points with significantly variable means of 1.27, 0.33, and 0.82 respectively (*p* < 0.001). We also identified statistically significant differences by centre. Scores for Centre A, the busier more urban site, were consistently lower than Centre B for all Pause Points. The most significant gap occurred upon admission (Pause Point 1) where Centre A averaged less one vital sign recorded (0.82, SD:1.01) and Centre B averaged 1.56 (SD:0.87) (*p* < 0.001). While scores for both centres for Pause Point 2 were less than 0.50, Centre B score higher with an average of 0.43 (SD: 0.81) vital statistics recorded compared to 0.25 (SD: 0.70) at Centre A (*p* < 0.05). No significant difference was found between centres for Pause Point 3 scores.

About 65% (*n* = 175) of maternal charts captured births that occurred in the post-intervention period at these two health centres and showed an increased likelihood in the documentation of heart rate/pulse and BP for the first and last Pause Points (Table 2). Overall, post-intervention vital sign measurement increased by 23%. This included a greater than twofold increase in the odds of reporting across heart rate/pulse and BP in both the unadjusted and adjusted models that accounted for centre-based discrepancies for Pause Points 1 and 3. However, in both the pre- and post-intervention periods, recording of care in the second Pause Point, immediately before birth, remained under 20%. Finally, even post-intervention, no variable was recorded more than 60% of the time for any given Pause Point.

**Table 1:**
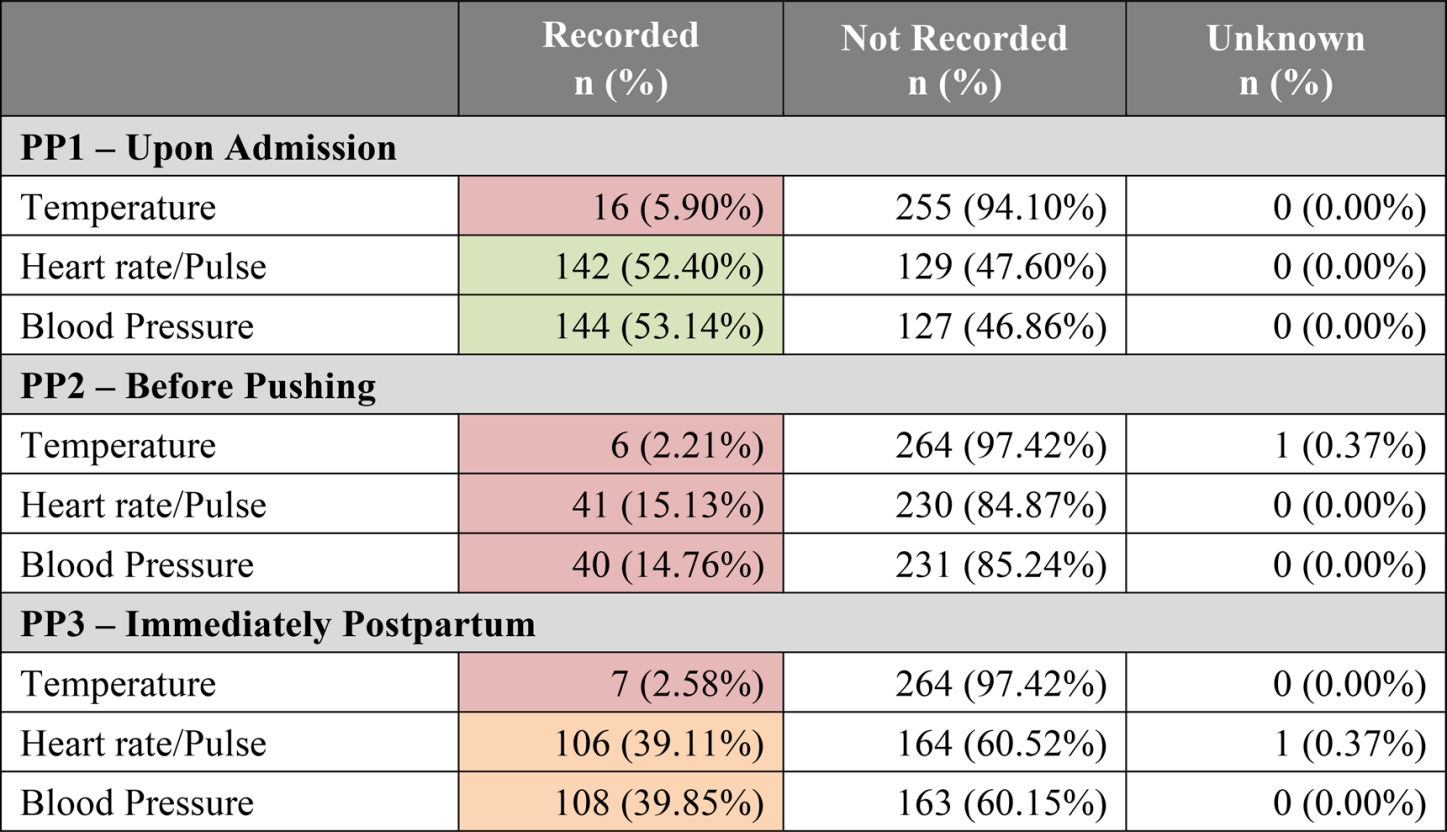
Pause Point (PP) Vital Signs Recorded at Two Health Centres (*N*=271) in Blantyre District Malawi

Shading: Green = recorded >50% of the time; Orange = recorded between 25–50% of the time; Red = recorded <25% of the time. *“All variables” summarizes the number and percentage of times temperature, heart rate/pulse, and blood pressure were all recorded vs. all not recorded. In some instances, 1 or 2 variables were recorded as evidenced by the sum of these percentages ≠ 100

**Table 2:** Recording of Key Vital Signs by Pause Point (PP) Pre-Intervention (*N* = 96) and Post-Intervention (*N* = 175)

	Pre-Int *n* (%)	Post-Int *n* (%)	UOR (95% CI)	AOR (95%CI) includes centre
PP1 - Admission				
**Temperature**			0.909 (0.327, 2.747)	0.913 (0.328, 2.762)
recorded	6 (6.2%)	10 (5.7%)		
not recorded	90 (93.8%)	165 (94.3%)		
**Heart rate/Pulse**			2.392 (1.442, 4.009)***	2.868 (1.655, 5.077)***
recorded	37 (38.5%)	105 (60.0%)		
not recorded	59 (61.5%)	70 (40.0%)		
**Blood Pressure**			2.192 (1.325, 3.660)**	2.728 (1.557, 4.890)***
recorded	39 (40.6%)	105 (60.0%)		
not recorded	57 (59.4%)	70 (40.0%)		
**PP2 - Before Pushing**				
**Temperature**			1.099 (0.211, 8.036)	1.113 (0.213, 8.146)
recorded	2 (2.1%)	4 (2.3%)		
not recorded	94 (97.9%)	171 (97.7%)		
**Heart rate/Pulse**			1.390 (0.688, 2.965)	1.418 (0.892, 3.427)
recorded	12 (12.5%)	29 (16.6%)		
not recorded	84 (87.5%)	146 (83.4%)		
**Blood Pressure**			1.165 (0.580, 2.444)	1.193 (0.590, 2.519)
recorded	13 (13.5%)	27 (15.4%)		
not recorded	83 (86.5%)	148 (84.6%)		
**PP3 - Immediately Postpartum**				
**Temperature**			0.725 (0.157, 3.746)	0.751 (0.161, 3.908)
recorded	3 (3.1%)	4 (2.3%)		
not recorded	93 (96.9%)	171 (97.7%)		
**Heart rate/Pulse**			2.103 (1.241, 3.632)**	2.125 (1.253, 3.677)**
recorded	27 (28.1%)	79 (45.1%)		
not recorded	69 (71.9%)	96 (54.9%)		
**Blood Pressure**			2.769 (1.617, 4.863)***	2.806 (1.635, 4.940)***
recorded	24 (25.0%)	84 (48.0%)		
not recorded	72 (75.0%)	91 (52.0%)		

p-values: *** >0.001; ** >0.01; * >0.05

While few maternal charts included all vital signs at each of the Pause Points, there was a significant increase in the number of vital signs recorded between the pre- and post-intervention periods (Table 3). Before the intervention, all vital signs were recorded for the Pause Points 1.0% to 3.1% of the time while after the intervention this range increased slightly to 1.7% to 5.1%. Likewise, the percentages of no vital signs recorded decreased for Pause Point 1 from 54% to 37% and Pause Point 3 from 72% to 51%. There was also a significant increase in the mean *Average Vital Statistics Recorded Score* for Pause Point 1, increasing from 0.85 (SD: 0.98) pre-intervention to 1.26 (SD:1.02) post-intervention (*p* < 0.01). Similarly, the mean score for Pause Point 3 significantly increased from 0.56 (SD: 0.94) pre-intervention to 0.95 (SD: 1.01) post-intervention (*p* < 0.01). There was no significant increase in Pause Point 2.

**Table 3:** Number of total key vital signs by pause point (PP) Pre-Intervention (*N* = 96) and Post-Intervention(*N* = 175)

	Total (*N* = 271)	Pre-Int (*N* = 96) *n* (%)	Post-Int (*N* = 175) *N* (%)	*p*-value
PP1 - Admission				
**all recorded**	11 (4.1%)	2 (2.1%)	9 (5.1%)	0.017
two recorded	125 (46.1%)	34 (35.4%)	91 (52.0%)	
one recorded	19 (7.01%)	8 (8.3%)	11 (6.3%)	
none recorded	116 (42.8%)	52 (54.2%)	64 (36.6%)	
**PP2 - Before Pushing**				
**all recorded**	4 (1.5%)	1 (1.0%)	3 (1.7%)	0.546
two recorded	34 (12.5%)	10 (10.4%)	24 (13.7%)	
one recorded	7 (2.6%)	4 (4.2%)	3 (1.7%)	
none recorded	226 (83.4%)	81 (84.4%)	145 (82.9%)	
**PP3 - Immediately Postpartum**				
**all recorded**	6 (2.2%)	3 (3.1%)	3 (1.7%)	0.002
two recorded	97 (35.8%)	21 (21.9%)	76 (43.4%)	
one recorded	9 (3.3%)	3 (3.1%)	6 (3.4%)	
none recorded	159 (58.7%)	69 (71.9%)	90 (51.4%)	

### Post-Intervention Sub-analyses

Recognizing the potential impact of the availability of supplies critical to the measurement of vital signs, sub-analyses explored the impact of the availability of key medical devices on documentation during the post-intervention period (*n* = 175). Descriptive statistics showed that functioning thermometers were reported to be available at the centres 77.6% (SD: 30.8) of the time across the data collection period whereas stethoscopes were available 37.1% (SD: 47.1) of the time and BP cuffs 99.0% (SD:4.4). Importantly, we found that medical devices used for vital sign measurement varied significantly by health centre. Most notably, the more rural centre, Centre A, never had a functioning stethoscope available whereas Centre B reported one for 95.6% (SD: 9.2) of the data collection period (*p* < 0.001). Availability of functioning BP cuffs were similarly high at 98.3% (SD: 5.6) of the data collection period in Centre A and 100% (SD: 0.0) in Centre B though statistically different (*p* < 0.05). Availability of functioning thermometers was 8.6% higher on average in Centre B compared to Centre A, though this difference was statistically insignificant.

When compared to maternal chart data, the recorded availability of thermometers and BP cuffs were not significantly associated with whether temperature or BP was recorded, respectively. Of note, we excluded stethoscope availability from multivariate analyses as it proved to be highly correlated with centre (Spearman’s rank correlation coefficient = 0.98). However, there was an association between the average number of recorded vital signs at the centres and the overall level of supplies available. For Pause Point 1, when the centres were not at all stocked or only partially stocked, no vital signs were recorded in 43 instances (24.57%). This was reduced by half to 21 instances (12.00%) of no vital signs recorded when the centres had availability of medical devices reviewed in this study (*p <* 0.01).There was no significant change observed in Pause Point 2 or 3.

## Discussion

We found statistically significant improvement in the documentation of maternal vital signs for the first and last SCC Pause Points during the period following the implementation of the intervention compared to the period prior. Importantly, this study relied on a retrospective review of patient charts and not direct observation of care, and thus the recording of Pause Points may not reflect the actual care received by the patient. Indeed, pre-dissemination discussions with stakeholders at the health centres, QECH, and with study team midwife mentors suggested that this can explain the lack of documentation for Pause Point 2. Collaborative review of our findings also shed light on the lower documentation of temperature compared to pulse and BP as an electric BP machine records both of the latter at once, while temperature must be manually taken by a provider.

Understaffing at the health centres in Blantyre results in significant and often conflicting demands on midwives’ time, particularly at the time of birth. When tasked with providing immediate care to a laboring mother and neonate (or multiple mothers and neonates at various stages of care), charting naturally takes second priority. However other studies, including those as part of GAIN in Malawi, have shown that mentorship increases not only documentation of, but also directly observed, QoC [14]. Accordingly, the increases seen in documentation at Pause Point one and three are likely to also represent an overall increase in the provision of that care. It is also worth noting that an increase in documentation on its own is important, as the results are key to informing the tracking of outcomes and thus allocation of vital resources.

Downstream issues stemming from understaffing are nuanced and the number of providers at any given centre only tells part of the story. While the global shortage of midwifery personnel has grown over the past decade, Malawi has nonetheless made tremendous improvements reaching more than 90% skilled attendance of births [8]. Despite this, as with other countries, Malawi continues to face challenges of provider burnout and workforce retention and motivation [6, 8, 21, 22]. Difficulties were exacerbated by indirect effects of the COVID-19 pandemic worsening shortages and reducing access to professional development opportunities [23]. While improving staffing infrastructure is needed at the systems-level in Malawi, QoC is also threatened by a lack of ongoing training to ensure existing staff have the confidence and skills to address complications [6]. In this way, the longitudinal mentorship component of the intervention appeared to prove beneficial despite shortages by scaling up current evidence-based approaches among staff, aligning with prior findings [14]. While not directly measured in our study, meaningfully supporting providers has also been shown to be critical to ensuring respectful care on the experience side of QoC [4, 24]. 

The discrepancies seen between health centres were not necessarily surprising. In low-resource settings such as Malawi, the social, cultural, and clinical norms of specific centres have been shown to have a greater impact on QoC than individual providers’ behavior change [25]. These differences are also often influenced by geographical and population divisions (i.e., rural vs. urban), with those proximal to desired amenities in urban areas able to recruit, retain, and support higher trained staff. In our study, the peri-urban site, known to be busier, had consistently lower documentation of vital signs. This relates directly to our earlier discussion of understaffing. We found that both the peri-urban and rural centre saw similar improvements in QoC indicators, despite significant centre-based differences. This suggests it is critical to provide the same level of training, support, and longitudinal mentorship across all centres.

Additionally, the role of supplies in the documentation of care is complex and demands more study. Although medical supplies are frequently tracked and quantified, our findings align with prior research stating that equipment is weakly related to QoC [26]. The peri-urban centre was frequently found to have one or more medical device(s) out of stock, which was suggested as due to providers from elsewhere in the centre borrowing supplies and not returning them. This naturally limits the ability of midwives to utilize those tools in the provision of care, and thus report the necessary vital signs. In our study, each centre was supported in the acquisition of critical medical devices for maternity care; however, their use as it pertains to QoC was unable to be captured in a maternal chart itself. For instance, while a functional BP cuff may be available, it also may be shared across multiple departments and not available for a given maternity patient or found during a site visit. In this way, both technological innovations and manual equipment that does not rely on power sources must be scaled up, supplemented with personnel training, and introduced with plans for sustainable, equitable use [27]. 

While our study sheds light on the benefits of longitudinal mentorship on a key aspect of maternal QoC, our approach was limited in a few ways. First, data collection was challenged given the retrospective nature of the study and local record-keeping practices. Women referred to tertiary care at QECH during labor, for instance, often had partial chart data at the primary centre which limited the number of charts with at least three SCC Pause Points recorded for analysis. Second, both quarterly and monthly stock checks varied in frequency—on average monthly checks were recorded two to five times per month per site. As a result, the number of checks in a particular month could have significantly impacted the availability of equipment recorded at that time. Related, while all mentors received the same training, interpersonal differences may have led to centre-specific differences that weren’t measured. We suspect the impact would be minor as findings aligned with expectations comparing a busier, urban site with a slower, rural one. Additionally, data collection for our study was limited to maternal partographs. This may explain our inability to analyze Pause Point 4 which we later learned is often documented separately in patients’ personal health passports that are not retained at the centres. Finally, given our pre-post study design, time may be a confounding factor though we do not perceive this had a significant impact on the findings. Still, we find confidence in our alignment with prior findings while acknowledging opportunities for further exploration.

A deeper dive into the bottlenecks of supplies and equipment needed for vital sign measurement and how it affects QoC indicators is warranted. Particularly in resource limited settings, including Malawi, it may be worth exploring a way to document when supplies are not available directly in patient charts to prevent assumptions of poor care from providers. In this way, weak associations with supplies may be nuanced with patient-level data. Alternatively, investigating innovative solutions to improve the availability of supplies and measure the impact on QoC may shed light on the true barriers, which may include staff shortages or low motivation, among others [6]. Additional research should also be conducted to explore appropriate management of complications as well as patient outcomes related to SCC Pause Point documentation in Malawi given the goal to improve QoC in their design [28]. 

Alongside continued rigorous research efforts, district-level support should be increased to address ongoing issues and prevent further strain on human and physical resources. To partially compensate for staffing shortages and prevent burnout of nurse-midwives, it would be helpful to monitor staff placement across health centres. Similarly, a communication system directly to the DNO should be built to facilitate real-time access to site supply needs. Anecdotally, we have learned that stockouts at local pharmacies can cause delays in accessing key clinical supplies preventing quality care. Supply needs, however, are uneven across sites and a way to facilitate effective redistribution from one centre to another could alleviate short-term stockouts as occasionally a particular centre has an abundance of a needed supply. Routine tracking of supplies by a designated staff at each centre and DNO-led mobilization of supplies may also reduce the expiration of resources with a set shelf-life while allowing pharmacies time to replenish. Additionally, ensuring that relevant decision-makers have timely information to assess and improve health care quality is essential to improving care and trust from the community [26]. Resourceful approaches to support existing infrastructure while investing in strengthening it continues to be needed to improve QoC.

## Conclusion

Continuing and expanding longitudinal and multipronged efforts that ensure adequate, qualified staffing and functioning supplies are critical to improving maternal care quality across low-resource settings. At the same time, developing creative tools for meaningful measurement will be key to making sustainable maternal QoC improvement [26]. Rigorous evaluations of these efforts will be critical to understanding which strategies are most effective. Analyses for additional aspects of this project are ongoing to look further at QoC indicators specific to complications identified as priority areas by the health centres. Together with this study’s findings, this work can be used to inform childbirth related QoC improvement efforts across Malawi and beyond.

## Supplementary Information

Supplementary Material 1: Appendix 1. Malawi Safe Childbirth Checklist – Adapted by UCSF Global Action in Nursing (GAIN) from the World Health Organization (WHO) Safe Childbirth Checklist (SCC)

## Data Availability

Data for this manuscript was made available to us by the National Health Sciences Research Committee (NHSRC), Queen Elizabeth Central Hospital (QECH) in Malawi, and the Malawi Ministry of Health. Data are not public available though may be requested from the NHSRC. Contact the corresponding author for additional information.
