# Supplementary material for: Evaluating the impact of a longitudinal mentorship intervention on the documentation of maternal vital signs in Blantyre district, Malawi
Source: BMC Pregnancy Childbirth. 2025 Dec 30;25:1350. doi: 10.1186/s12884-025-08493-0 (PMC12752263; doi:10.1186/s12884-025-08493-0)
Supplement: Supplementary file 1 — Supplementary Material 1: Appendix 1. Malawi Safe Childbirth Checklist – Adapted by UCSF Global Action in Nursing (GAIN) from the World Health Organization (WHO) Safe Childbirth Checklist (SCC) [file 12884_2025_8493_MOESM1_ESM.pdf]

Malawi Safe Childbirth Checklist

GAIN Global Action in Nursing

Adapted from the World Health Organization Safe Childbirth Checklist

1 On Admission

Does mother need referral?

❑ No

❑ Yes, organized
Check your facility's criteria

Partograph started?

❑ No, will start when ≥ 4cm

❑ Yes
Start plotting when cervix ≥4 cm, then cervix should dilate ≥1 cm/hr
- Every 30 min: assess and record fetal heart rate
- Every hour: assess and record maternal heart rate, blood pressure, contractions and descent
- Every 2 hours: assess and record temperature
- Every 4 hours: perform vaginal examination

Does mother have signs of infection? Assess for *antibiotics* or *antimalarials*.

❑ No

❑ Yes, ANTIBIOTICS given
Ask for allergies before administration of any medication
Give antibiotics to mother if any of the following presents:
- Mother's temperature ≥38°C
- Foul-smelling vaginal discharge
- Rupture of membranes >18 hrs

❑ Yes, ANTIMALARIALS given
- Perform Malaria Rapid Test if mother has fever, headache, malaise, tachycardia

Does mother need *Magnesium Sulphate* and *antihypertensive treatment*?

❑ No, blood pressure is normal

❑ Yes, MAGNESIUM SULPHATE started
Give magnesium sulphate to mother if any of following present:
- Eclampsia
- Severe hypertension (more than one reading of either systolic BP ≥160 or diastolic BP≥110
- Hypertension and signs of impending eclampsia (severe headache, visual disturbance, epigastric pain)
- Proteinuria is NOT mandatory for preeclampsia diagnosis

Refer to protocols on initiation and administration of Magnesium Sulphate

❑ Yes, ANTIHYPERTENSIVE medication given
Give antihypertensive medication if severe hypertension (more than one reading of either systolic BP ≥160 or diastolic BP ≥110)
Goal: keep BP < 150 / 100 mmHg

Refer to protocols on Management of Severe Hypertension; IV medication may be recommended

Referral or consultation is required for patients requiring antihypertensive medications

What is the mother's HIV serostatus?

❑ Negative, performed within the last 3 months

❑ Negative, performed more than 3 months ago: repeat rapid test now

❑ Positive on ART

❑ Positive, not on ART: start ART now, as per protocol

Is gestational age greater than 37 weeks, using best available dating criteria?

❑ No or unsure
- Perform ultrasound if available
- Start IV line and fluids, perform FBC and urinalysis if available
- Mother may benefit from tocolysis and dexamethasone, refer to protocols

❑ Yes
- Continue with routine management

Confirm supplies are available to clean hands and wear gloves for each vaginal exam.

Encourage birth companion to be present at birth.

Confirm that mother or companion will call for help during labour if needed:
▪ Bleeding ▪ Severe abdominal pain ▪ Severe headache or visual disturbance ▪ Unable to urinate ▪ Urge to push

GAIN Global Action in Nursing
Adapted from the World Health Organization Safe Childbirth Checklist

2 Just Before Pushing (Or Caesarean)

Does mother have signs of infection? Assess for *antibiotics* or *antimalarials*.

❑ No
❑ Yes, ANTIBIOTICS given
Ask for allergies before administration of any medication
Give antibiotics to mother if any of the following presents:

- Mother's temperature ≥38 °C
- Foul-smelling vaginal discharge
- Rupture of membranes >18 hrs
- Caesarean section

❑ Yes, ANTIMALARIALS given

- Fever that develops in labour is more likely an ascending infection; less likely to be malaria
- Perform Malaria Rapid Test if mother has fever, headache, malaise, tachycardia

Does mother need *Magnesium Sulphate* and *antihypertensive treatment*?

❑ No, blood pressure is normal
❑ Yes, MAGNESIUM SULPHATE started
Give magnesium sulphate to mother if any of following present:

- Eclampsia
- Severe hypertension (more than one reading of either systolic BP ≥160 or diastolic BP≥110
- Hypertension and signs of impending eclampsia (severe headache, visual disturbance, epigastric pain)
- Proteinuria is NOT mandatory for preeclampsia diagnosis

Refer to protocols on initiation and administration of Magnesium Sulphate

❑ Yes, ANTIHYPERTENSIVE medication given
Give antihypertensive medication if severe hypertension (more than one reading of either systolic BP ≥160 or diastolic BP ≥110)
Goal: keep BP < 150 / 100 mmHg

Refer to protocols on Management of Severe Hypertension; IV medication may be recommended

Referral or consultation is required for patients requiring antihypertensive medications

Confirm essential supplies are at bedside and prepare for delivery:

For mother:
❑ Gloves
❑ Alcohol-based handrub or soap and clean water
❑ Oxytocin 10 units in syringe

For baby:
❑ Clean towel
❑ Tie or cord clamp
❑ Sterile blade to cut cord
❑ Suction device
❑ Bag-and-mask

Assistant identified and ready to help at birth if needed.

Prepare to care for mother immediately after birth:

- Confirm single baby only (not multiple birth)
- Give oxytocin within 1 minute after birth
- Deliver placenta with controlled cord traction. Consider manual removal after 30 minutes, or sooner if evidence of postpartum hemorrhage
- Massage uterus after placenta is delivered
- Confirm uterus is well-contracted

Prepare to care for baby immediately after birth:

Dry baby, keep warm.
If infant born vigorous, delay cord clamping for 2-3 minutes or until pulsations have ceased.
If not breathing, stimulate and clear airway.
If still not breathing:

- clamp and cut cord
- clean airway if necessary
- ventilate with bag-and-mask
- shout for help

GAIN Global Action in Nursing

Adapted from the World Health Organization Safe Childbirth Checklist

3 Soon After Birth (Within 1 Hour)

Is mother bleeding abnormally?

❑ No

❑ Yes, shout for help

- Massage uterus
- Check vital signs (pulse, blood pressure)
- Consider more uterotonic (40 units oxytocin in 1 litre normal saline, or misoprostol per protocol, if available)
- Start IV and keep mother warm
- Catherize bladder
- Treat cause: uterine atony, retained placenta/fragments, vaginal tear, uterine rupture
- Consider placing NASG if signs of shock are present

Does mother have signs of infection? Assess for *antibiotics* or *antimalarials*.

❑ No

❑ Yes, ANTIBIOTICS given

Ask for allergies before administration of any medication

Give antibiotics to mother if any of the following presents:

- Mother's temperature ≥38 °C
- Foul-smelling vaginal discharge
- Rupture of membranes >18 hrs
- Caesarean section, manual removal of placenta, or third/fourth degree tear

❑ Yes, ANTIMALARIALS given

- Fever that develops in labour is more likely an ascending infection; less likely to be malaria
- Perform Malaria Rapid Test if mother has fever, headache, malaise, tachycardia

Does mother need *Magnesium Sulphate* and *antihypertensive treatment*?

❑ No, blood pressure is normal

❑ Yes, MAGNESIUM SULPHATE started

Give magnesium sulphate to mother if any of following present:

- Eclampsia
- Severe hypertension (more than one reading of either systolic BP ≥160 or diastolic BP≥110
- Hypertension and signs of impending eclampsia (severe headache, visual disturbance, epigastric pain)
- Proteinuria is NOT mandatory for preeclampsia diagnosis

Refer to protocols on initiation and administration of Magnesium Sulphate

❑ Yes, ANTIHYPERTENSIVE medication given

Give antihypertensive medication if severe hypertension (more than one reading of either systolic BP ≥160 or diastolic BP ≥110)

Goal: keep BP < 150 / 100 mmHg

Refer to protocols on Management of Severe Hypertension; IV medication may be recommended

Referral or consultation is required for patients requiring antihypertensive medications

Does baby need: *referral?*

❑ No

❑ Yes, organized

- Check your facility's criteria

Does baby need: *antibiotics?*

❑ No

❑ Yes, given

Give baby antibiotics if antibiotics given to mother for treatment of maternal infection during childbirth or if baby has any of:

- Respiratory rate >60/min or <30/min
- Chest in-drawing, grunting, or convulsions
- Poor movement on stimulation
- Baby's temperature <35 °C (and not rising after warming) or baby's temperature ≥38 °C

Does baby need: *special care and monitoring?*

❑ No

❑ Yes, organized

Refer baby to nursery care if any of the following is present:

- More than 1 month early
- Birth weight <2500 grams
- Needs antibiotics
- Required resuscitation

Started breastfeeding and skin-to-skin contact (if mother and baby are well).

Keep skin-to-skin for at least one hour if able; within one hour of life, administer routine newborn medications per protocol:

▪ **Vitamin K** injection ▪ **Tetracycline** eye ointment ▪ **Chlorhexidine** for cord care ▪ **Nevirapine** syrup, if applicable

Confirm mother / companion will call for help if danger signs present.

4 Before Discharge

Confirm stay at facility for 48 hours after delivery.

For severely overcrowded facilities, consider **24-hour minimum only** for multiparous women with no complications.

Does mother have signs of infection? Assess for *antibiotics* or *antimalarials*.

❑ No

❑ Yes, ANTIBIOTICS given and discharge delayed

Ask for allergies before administration of any medication

Give antibiotics to mother if any of the following presents:

- Mother's temperature ≥38 °C
- Foul-smelling vaginal discharge
- Not adequately treated for other obstetric indications

i.e. Caesarean section, manual removal of placenta, or third/fourth degree tear

❑ Yes, ANTIMALARIALS given and discharge delayed

- Perform Malaria Rapid Test if mother has fever, headache, malaise, tachycardia

Does mother need to start: magnesium sulfate or antihypertensive treatment?

❑ No, blood pressure is normal

❑ Yes, MAGNESIUM SULPHATE started and discharge delayed

Give magnesium sulphate to mother if any of following present:

- Eclampsia
- Severe hypertension (more than one reading of either systolic BP ≥160 or diastolic BP≥110
- Hypertension and signs of impending eclampsia (severe headache, visual disturbance, epigastric pain)
- Proteinuria is NOT mandatory for preeclampsia diagnosis

Refer to protocols on initiation and administration of Magnesium Sulphate

❑ Yes, ANTIHYPERTENSIVE medication given and discharge delayed

Give antihypertensive medication if severe hypertension (more than one reading of either systolic BP ≥160 or diastolic BP ≥110)

Goal: keep BP < 150 / 100 mmHg

Refer to protocols on Management of Severe Hypertension; IV medication may be recommended

Referral or consultation is required for patients requiring antihypertensive medications

Is mother bleeding abnormally?

❑ No

❑ Yes, treat and delay discharge; consider referral

Does baby need to start antibiotics?

❑ No

❑ Yes, given and discharge delayed

Give baby antibiotics if antibiotics given to mother for treatment of maternal infection during childbirth or if baby has any of:

- Respiratory rate >60/min or <30/min
- Chest in-drawing, grunting, or convulsions
- Poor movement on stimulation
- Baby's temperature <35 °C (and not rising after warming) or baby's temperature ≥38 °C

Is baby feeding well?

❑ No, delay discharge

- Observe baby breastfeeding if possible
- Weigh baby before discharge and hold discharge if weight loss is >10%

❑ Yes, based on weight and observation

Discuss and offer family planning options to mother.

Consider administration prior to discharge for high risk mother

Arrange follow-up and confirm mother / companion will seek help if danger signs appear after discharge.

Review Nevirapine administration and PMTCT follow-up, if applicable.

Danger Signs

MOTHER has any of:

- Bleeding
- Severe abdominal pain
- Severe headache or visual disturbance
- Breathing difficulty
- Fever or chills
- Difficulty emptying bladder
- Epigastric pain

BABY has any of:

- Fast/difficult breathing
- Fever
- Unusually cold
- Stops feeding well
- Whole body becomes yellow
- Less activity than normal
